# Disseminated Eruptive Blue Nevi in a Young Adult Patient

**DOI:** 10.7759/cureus.11298

**Published:** 2020-11-02

**Authors:** Hasina Maredia, Amarachi Eseonu, Sima Rozati

**Affiliations:** 1 Department of Dermatology, Johns Hopkins University School of Medicine, Baltimore, USA

**Keywords:** eruptive blue nevi, benign nevi, melanocytes

## Abstract

Common blue nevi tend to be singular or localized, with multiple eruptive blue nevi being a rare occurrence. We report the case of a young adult who presented with multiple asymptomatic lesions that had appeared gradually over a few years. Physical examination revealed 30 distinct, blue-gray macules diffusely over the medial buttocks, lower back, and dorsal arms. Histopathology showed pigmented dendritic melanocytes with associated melanophages, features characteristic of blue nevus. This case demonstrates that eruptive blue nevi can present as numerous, disseminated lesions over multiple anatomic sites. Recognition of the various patterns of eruptive blue nevi and their benign nature can reduce unnecessary biopsies and work-up.

## Introduction

Common blue nevi occur in about 0.5-4.0% of healthy white adults, while multiple eruptive blue nevi are rarer and have been described in only 13 case reports so far [1-7]. These cases were associated with a known or possible trigger, and only two of them had multiple areas of the body involved [2,6]. We present a unique case of disseminated, discrete eruptive blue nevi over multiple anatomic sites to highlight a rare but benign presentation.

## Case presentation

**Figure 1:**
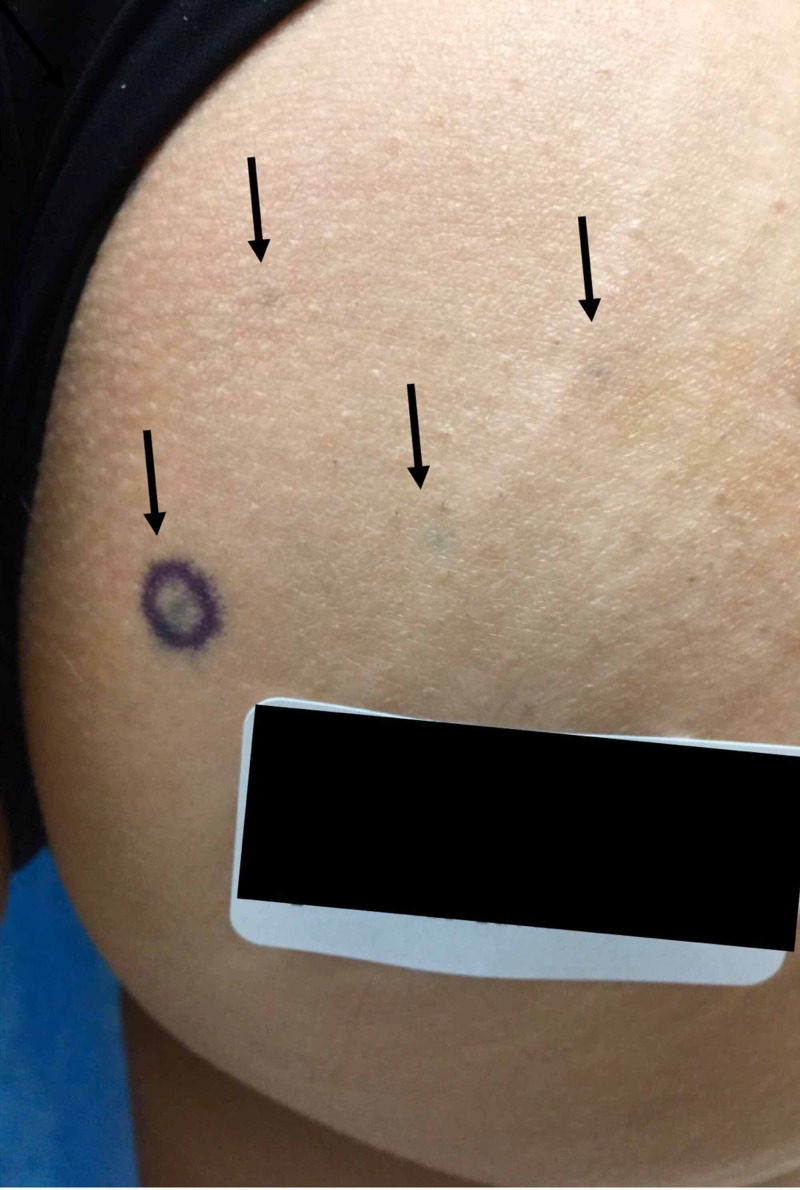
Clinical image of the right buttock demonstrating blue-gray macules Four 1-3 mm, blue-gray, and well-demarcated macules on the right medial buttock pictured. The patient had additional similar macules on the left buttock, lower back, and bilateral dorsal arms, totaling around 30 macules

**Figure 2:**
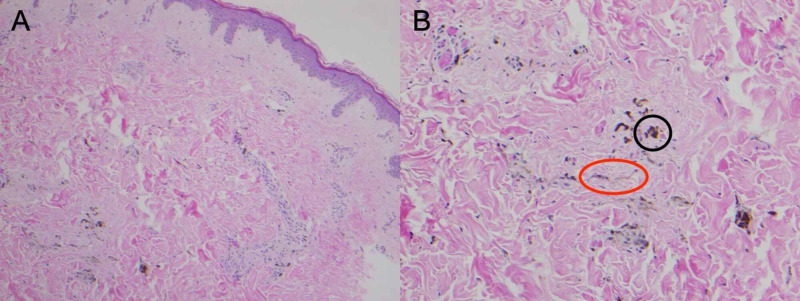
Punch biopsy specimen from lesion on the right buttock Ill-defined, deep dermal proliferation of pigmented dendritic melanocytes (red circle) with associated melanophages (black circle). No associated mitoses or necrosis were appreciated, consistent with common blue nevus. Hematoxylin-eosin (A) x10 (B) x20

A young adult presented to dermatology with multiple, discrete round small blue-gray macules that had first appeared a few years ago. The patient was otherwise healthy except for a history of scoliosis. The macules had initially appeared on the bilateral buttocks and then on the lower back and arms. The lesions were asymptomatic and there was no reported trauma to the area nor any tanning-bed use. There was no family history of similar lesions. On exam, 30 distinct, blue-gray, and well-demarcated macules, ranging in size from 1 mm to 3 mm, were found on the medial buttocks (Figure 1), lower back, and bilateral dorsal arms. A punch biopsy was performed, and histopathology revealed pigmented dendritic melanocytes with associated melanophages, consistent with blue nevus (Figure 2).

## Discussion

Diagnosis of blue nevi can be made clinically, although atypical and challenging cases may require a biopsy to confirm the diagnosis. Histologically, the blue nevi are located in the mid-dermis and are thought to arise from dendritic melanocytes that have not migrated to the epidermis from their site of origin in neural crest cells [1-4]. They commonly present in areas with residual dermal melanocytes, including the scalp, pre-sacrum, and dorsal extremities [5-7].

Types of blue nevi, based on clinical and histopathological differences, include common and cellular [1-3]. Common blue nevi tend to be solitary blue-black papules, less than 10 mm in size. Histologically, they appear as an admixture of pigmented dendritic melanocytes and melanophages in a fibrotic stroma [3]. In contrast, cellular blue nevi are firm, blue-black, and dome-shaped nodules or plaques that tend to be 1-2 cm in size but can be larger [5]. Histologically, cellular blue nevi are characterized by distinct cellular areas with spindled-to-oval-shaped melanocytes and clear-to-lightly pigmented cytoplasm [3].

Among the 13 cases of eruptive blue nevi reported so far, only two had multiple areas involved, namely a patient with bilateral tibial involvement and a second with diffuse distribution over the body [2,6]. However, both had a history of additional cutaneous lesions. The former had cutis marmorata telangiectatica congenita along with limb hypoplasia [6]. The latter had generalized congenital dermal melanocytosis and acquired bilateral nevus of Ota, and the patient's blue nevi erupted after starting tanning-bed use and oral contraceptives [2]. In comparison, our patient presented uniquely with a disseminated distribution of discrete multiple blue nevi and no associated lesions.

The cases of eruptive blue nevi were associated with a known or possible trigger, although there may have been a selection bias for reporting such cases. Triggers or associations have included trauma, sunburns, oral contraceptives, and vesiculobullous dermatoses [1-3]. In addition, multiple blue nevi may also be associated with the lentigines, atrial myxomas, mucocutaneous myxomas, and blue nevi (LAMB) syndrome [3]. Our patient did not have any identifiable trigger or association.

It is important to distinguish blue nevi from satellite metastatic melanoma or regressive melanoma [4]. Common blue nevi are stable and can regress, though there have been rare reports of malignant blue nevi, mostly with the cellular type [3]. Thus, the presence of nodules or changes in the appearance of blue nevi warrants work-up. Other differentials for blue nevi include glomus tumor, Spitz nevi, sclerosing hemangioma, Kaposi sarcoma, and dermatofibroma [2]. Atypical acquired dermal melanocytoses are also on the differential, but a review of 15 such cases found that they occur mainly over the face, extremities, and the back with only three cases that had melanophages on histology [8]. Thus, our case was most consistent clinically and histologically with eruptive blue nevi.

## Conclusions

Given limited data in the literature, our case report provides an example of eruptive blue nevi that can be benign even when numerous, disseminated over multiple anatomic sites, and sudden in appearance. The recognition of unique, benign patterns can reduce unnecessary biopsies and provide reassurance to patients. Further long-term follow-up studies are needed to truly assess the risk of malignant transformation.
